# *trans*-Bis[8-(benzyl­sulfan­yl)quinoline-κ^2^ *N*,*S*]di­chlorido­cobalt(II)

**DOI:** 10.1107/S2414314621009925

**Published:** 2021-10-04

**Authors:** Shintaro Kodama, Kazuki Bunno, Akihiro Nomoto, Akiya Ogawa

**Affiliations:** a Department of Applied Chemistry, Graduate School of Engineering, Osaka, Prefecture University, 1-1 Gakuen-cho, Nakaku, Sakai, Osaka 599-8531, Japan; Purdue University, USA

**Keywords:** crystal structure, cobalt(II) complex, bidentate *N*,*S*-ligand

## Abstract

The title di­chloro­cobalt(II) complex, *trans*-[CoCl_2_(**1**)_2_] [**1** = 8-(benzyl­sulfanyl)­quinoline, C_16_H_13_NS], has a central Co^II^ atom (site symmetry  ) that exhibits a distorted octa­hedral coordination geometry and is coordinated by two N and two S atoms from the bidentate *N*,*S*-ligand (**1**) situated in an equatorial plane and two Cl atoms in the axial positions. Complexes are linked by weak inter­molecular C—H⋯π inter­actions between the 8-(benzyl­sulfanyl)­quinoline ligands, forming a chain extending along the *a*-axis direction.

## Abstract 2

The title di­chloro­cobalt(II) complex has a Co^II^ center that exhibits a distorted octa­hedral coordination geometry and is coordinated by two N and two S atoms from the bidentate *N*,*S*-ligand situated in the equatorial plane and two Cl atoms in the axial positions.

## Graphical abstract

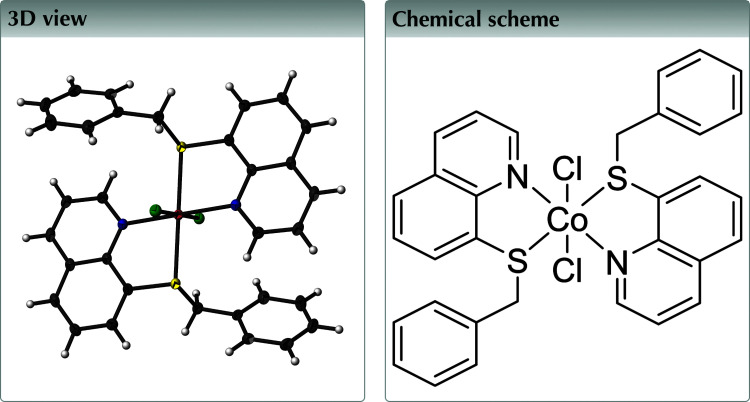

## Structure description

Di­chlorido­cobalt(II) complexes with homo donor ligands (*e.g.*, multidentate nitro­gen ligands) have been widely used in catalytic applications (Ma *et al.*, 2014 ▸; Ai *et al.*, 2019 ▸; Guo *et al.*, 2021 ▸). Di­chlorido­cobalt(II) complexes with hetero donor ligands (*e.g.*, nitro­gen- and sulfur-containing multidentate ligands) also exhibit inter­esting catalytic activities, *e.g.* in the oxidation reaction of *n*-octane (Soobramoney *et al.*, 2014 ▸) and in the photochemical-driven hydrogen evolution from water (Lei *et al.*, 2018 ▸); however, they are still limited in number. Herein, we report the structure determination of a new di­chlorido­cobalt(II) complex **2** with 8-(benzyl­sulfanyl)­quinoline (**1**) as an *N*,*S*-ligand (Kita *et al.*, 2002 ▸) by single-crystal X-ray analysis.

**Figure 1:**
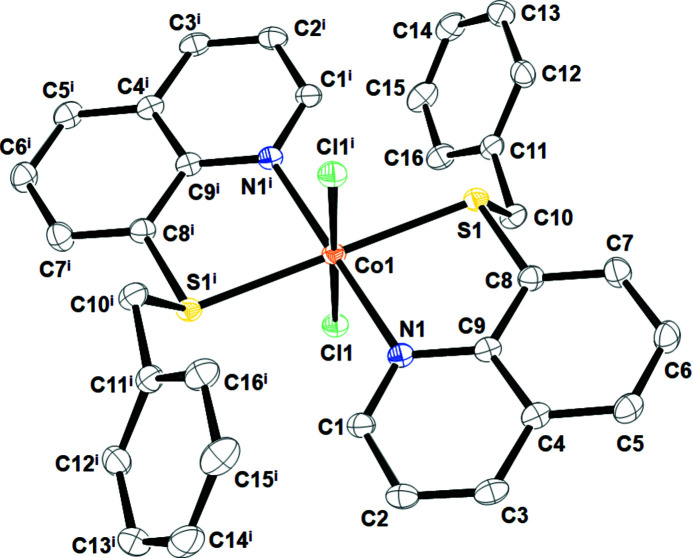
The mol­ecular structure of **2** with atom numbering. Dispacement ellipsoids are drawn at the 50% probability level. H atoms are omitted for clarity. [Symmetry code: (i) 1 − *x*, 1 − *y*, 1 − *z*.]

**Figure 2:**
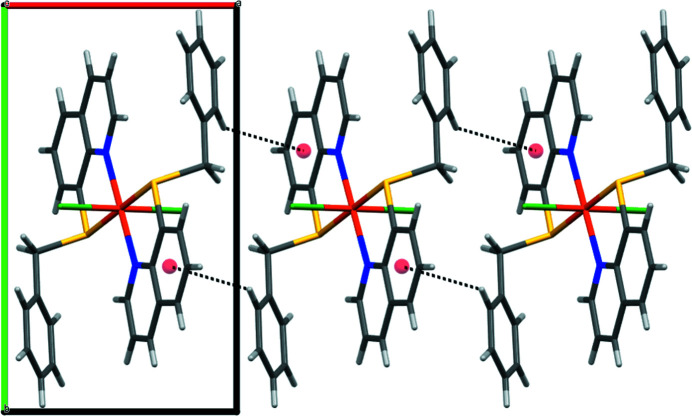
Crystal packing of **2** viewed along the *c* axis.

As presented in Fig. 1 ▸, complex **2** exhibits a distorted octa­hedral coordination geometry. The central Co^II^ atom, located on a crystallographic center of inversion, is coordinated by two N and two S atoms from two symmetry-equivalent ligands **1** situated in the equatorial plane and two Cl atoms in the axial positions. The Co—N [2.1543 (17) Å] and Co—S [2.4856 (5) Å] bond lengths are within the range of those found in di­chlorido­cobalt(II) complexes with a nitro­gen- and sulfur-containing multidentate ligand (Soobramoney *et al.*, 2014 ▸; Lei *et al.*, 2018 ▸). In addition, weak inter­molecular C—H⋯π inter­actions between the 8-(benzyl­sulfanyl)­quinoline ligands are observed in the crystal packing of **2** (Karle *et al.*, 2007 ▸), forming a chain along the *a*-axis direction (Fig. 2 ▸ and Table 1 ▸).

## Synthesis and crystallization

CoCl_2_·6H_2_O (18.5 mg, 0.078 mmol) and 8-(benzyl­sulfanyl)­quinoline (**1**; 45.5 mg, 0.18 mmol) in EtOH (20 mL) were heated at reflux overnight. The solvents were evaporated from the resulting suspension, and the residue was suspended in Et_2_O followed by filtration to obtain a yellow–green powder. The powder was dissolved in EtOH, and Et_2_O was diffused into the resulting solution to give **2** (4.5 mg, 9% yield) as yellow crystals. M.p. 150.2–150.8°C; IR (KBr, cm^−1^) 3045, 2998, 1595, 1493, 1452, 1371, 1312, 1240, 994, 833.

## Refinement

Crystal data, data collection and structure refinement details are summarized in Table 2 ▸.

## Supplementary Material

Crystal structure: contains datablock(s) I. DOI: 10.1107/S2414314621009925/zl4046sup1.cif

Structure factors: contains datablock(s) I. DOI: 10.1107/S2414314621009925/zl4046Isup2.hkl

CCDC reference: 2111388

Additional supporting information:  crystallographic information; 3D view; checkCIF report

## Figures and Tables

**Table 1:** Hydrogen-bond geometry (Å, °) *Cg*2 is the centroid of the C4–C9 ring.

*D*—H⋯*A*	*D*—H	H⋯*A*	*D*⋯*A*	*D*—H⋯*A*
C16—H16⋯*Cg*2^i^	0.95	2.89	3.575 (2)	131

Symmetry code: (i) 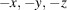 .

**Table 2:** Experimental details

Crystal data	
Chemical formula	[CoCl_2_(C_16_H_13_NS)_2_]
*M*_r_	632.50
Crystal system, space group	Monoclinic, *P*2_1_/*c*
Temperature (K)	103
*a*, *b*, *c* (Å)	8.00610 (17), 13.5141 (3), 13.3349 (3)
β (°)	105.714 (7)
*V* (Å^3^)	1388.85 (7)
*Z*	2
Radiation type	Mo *K*α
μ (mm^−1^)	0.99
Crystal size (mm)	0.09 × 0.04 × 0.02

Data collection	
Diffractometer	Rigaku VariMax RAPID
Absorption correction	Multi-scan (*ABSCOR*; Rigaku, 1995 ▸)
*T*_min_, *T*_max_	0.780, 1.000
No. of measured, independent and observed [*I* > 2σ(*I*)] reflections	22957, 3191, 2814
*R*_int_	0.034
(sin θ/λ)_max_ (Å^−1^)	0.649

Refinement	
*R*[*F* ^2^ > 2σ(*F* ^2^)], *wR*(*F* ^2^), *S*	0.031, 0.082, 1.18
No. of reflections	3191
No. of parameters	178
H-atom treatment	H-atom parameters constrained
Δρ_max_, Δρ_min_ (e Å^−3^)	0.44, −0.23

Computer programs: *RAPID-AUTO* (Rigaku, 1995 ▸); *SHELXT2018/2* (Sheldrick, 2015*a* ▸), *SHELXL2018/3* (Sheldrick, 2015*b* ▸) and *OLEX2* (Dolomanov *et al.*, 2009 ▸).

## full crystallographic data

### Crystal data

**Table:**

[CoCl_2_(C_16_H_13_NS)_2_]	*F*(000) = 650
*M**_r_* = 632.50	*D*_x_ = 1.512 Mg m^−^^3^
Monoclinic, *P*2_1_/*c*	Mo *K*α radiation, λ = 0.71075 Å
*a* = 8.00610 (17) Å	Cell parameters from 18593 reflections
*b* = 13.5141 (3) Å	θ = 2.2–27.5°
*c* = 13.3349 (3) Å	µ = 0.99 mm^−^^1^
β = 105.714 (7)°	*T* = 103 K
*V* = 1388.85 (7) Å^3^	Prism, colourless
*Z* = 2	0.09 × 0.04 × 0.02 mm

### Data collection

**Table:**

Rigaku VariMax RAPID diffractometer	3191 independent reflections
Radiation source: rotating anode X-ray generator, MicroMax 007	2814 reflections with *I* > 2σ(*I*)
Multi-layer mirror optics monochromator	*R*_int_ = 0.034
Detector resolution: 10.0 pixels mm^-1^	θ_max_ = 27.5°, θ_min_ = 2.6°
ω scans	*h* = −9→10
Absorption correction: multi-scan (ABSCOR; Rigaku, 1995)	*k* = −17→17
*T*_min_ = 0.780, *T*_max_ = 1.000	*l* = −17→17
22957 measured reflections	

### Refinement

**Table:**

Refinement on *F*^2^	Primary atom site location: dual
Least-squares matrix: full	Hydrogen site location: inferred from neighbouring sites
*R*[*F*^2^ > 2σ(*F*^2^)] = 0.031	H-atom parameters constrained
*wR*(*F*^2^) = 0.082	*w* = 1/[σ^2^(*F*_o_^2^) + (0.0232*P*)^2^ + 1.8429*P*] where *P* = (*F*_o_^2^ + 2*F*_c_^2^)/3
*S* = 1.18	(Δ/σ)_max_ < 0.001
3191 reflections	Δρ_max_ = 0.44 e Å^−^^3^
178 parameters	Δρ_min_ = −0.23 e Å^−^^3^
0 restraints	

### Special details

**Table:**

Geometry. All esds (except the esd in the dihedral angle between two l.s. planes) are estimated using the full covariance matrix. The cell esds are taken into account individually in the estimation of esds in distances, angles and torsion angles; correlations between esds in cell parameters are only used when they are defined by crystal symmetry. An approximate (isotropic) treatment of cell esds is used for estimating esds involving l.s. planes.
Refinement. H atoms were positioned geometrically and constrained to ride on their parent atoms with C—H and CH_2_ bond distances of 0.95 and 0.99 Å. *U*_iso_(H) values were set to 1.2 *U*_eq_(C).

### Fractional atomic coordinates and isotropic or equivalent isotropic displacement parameters (Å^2^)

**Table:**

	*x*	*y*	*z*	*U*_iso_*/*U*_eq_	
Co1	0.500000	0.500000	0.500000	0.01456 (10)	
Cl1	0.75883 (6)	0.50541 (4)	0.64296 (4)	0.01844 (12)	
S1	0.64306 (7)	0.43574 (4)	0.36942 (4)	0.01617 (12)	
N1	0.5642 (2)	0.63898 (13)	0.44057 (13)	0.0157 (3)	
C1	0.5280 (3)	0.72466 (16)	0.47761 (17)	0.0182 (4)	
H1	0.479481	0.723445	0.535214	0.022*	
C2	0.5564 (3)	0.81773 (16)	0.43738 (18)	0.0203 (4)	
H2	0.527515	0.876968	0.467243	0.024*	
C3	0.6260 (3)	0.82107 (16)	0.35484 (17)	0.0205 (4)	
H3	0.646246	0.882891	0.326311	0.025*	
C4	0.6677 (3)	0.73190 (16)	0.31209 (16)	0.0172 (4)	
C5	0.7444 (3)	0.73145 (17)	0.22805 (17)	0.0211 (4)	
H5	0.765834	0.792080	0.197652	0.025*	
C6	0.7877 (3)	0.64363 (17)	0.19073 (17)	0.0227 (5)	
H6	0.841301	0.643561	0.135286	0.027*	
C7	0.7535 (3)	0.55343 (17)	0.23382 (17)	0.0219 (5)	
H7	0.781298	0.492970	0.205753	0.026*	
C8	0.6802 (3)	0.55136 (15)	0.31611 (16)	0.0173 (4)	
C9	0.6357 (3)	0.64133 (15)	0.35748 (16)	0.0159 (4)	
C10	0.8657 (3)	0.39310 (16)	0.42715 (18)	0.0196 (4)	
H10A	0.936644	0.403595	0.377737	0.023*	
H10B	0.918744	0.430657	0.491654	0.023*	
C11	0.8590 (3)	0.28453 (16)	0.45155 (17)	0.0183 (4)	
C12	0.8020 (3)	0.21507 (17)	0.37238 (19)	0.0231 (5)	
H12	0.769255	0.235779	0.301657	0.028*	
C13	0.7929 (3)	0.11537 (17)	0.3968 (2)	0.0271 (5)	
H13	0.752454	0.068236	0.342799	0.033*	
C14	0.8429 (3)	0.08496 (17)	0.4998 (2)	0.0280 (5)	
H14	0.836682	0.016931	0.516390	0.034*	
C15	0.9017 (3)	0.15333 (18)	0.5785 (2)	0.0277 (5)	
H15	0.938461	0.132049	0.649012	0.033*	
C16	0.9073 (3)	0.25348 (17)	0.55467 (19)	0.0230 (5)	
H16	0.944155	0.300590	0.609058	0.028*	

### Atomic displacement parameters (Å^2^)

**Table:**

	*U*^11^	*U*^22^	*U*^33^	*U*^12^	*U*^13^	*U*^23^
Co1	0.0166 (2)	0.01190 (19)	0.01521 (19)	−0.00096 (15)	0.00437 (15)	0.00003 (15)
Cl1	0.0184 (2)	0.0177 (2)	0.0182 (2)	−0.00075 (19)	0.00324 (19)	−0.00025 (19)
S1	0.0184 (2)	0.0127 (2)	0.0175 (2)	0.00018 (19)	0.00518 (19)	0.00035 (19)
N1	0.0154 (8)	0.0149 (8)	0.0161 (8)	−0.0004 (7)	0.0030 (7)	0.0004 (7)
C1	0.0182 (10)	0.0162 (10)	0.0196 (10)	−0.0017 (8)	0.0044 (8)	−0.0016 (8)
C2	0.0204 (10)	0.0139 (10)	0.0251 (11)	0.0007 (8)	0.0034 (9)	−0.0012 (8)
C3	0.0194 (10)	0.0151 (10)	0.0235 (11)	−0.0016 (8)	0.0001 (9)	0.0039 (8)
C4	0.0161 (10)	0.0166 (10)	0.0164 (10)	−0.0009 (8)	0.0001 (8)	0.0034 (8)
C5	0.0235 (11)	0.0207 (11)	0.0175 (10)	−0.0022 (9)	0.0028 (8)	0.0064 (8)
C6	0.0270 (11)	0.0242 (11)	0.0184 (11)	0.0002 (9)	0.0088 (9)	0.0045 (9)
C7	0.0262 (11)	0.0206 (11)	0.0203 (10)	0.0032 (9)	0.0084 (9)	0.0014 (9)
C8	0.0192 (10)	0.0161 (10)	0.0159 (10)	−0.0006 (8)	0.0035 (8)	0.0018 (8)
C9	0.0154 (9)	0.0151 (9)	0.0157 (10)	0.0000 (8)	0.0014 (8)	0.0020 (8)
C10	0.0168 (10)	0.0161 (10)	0.0260 (11)	−0.0003 (8)	0.0061 (9)	0.0018 (9)
C11	0.0151 (10)	0.0155 (10)	0.0255 (11)	0.0022 (8)	0.0075 (8)	0.0014 (8)
C12	0.0289 (12)	0.0191 (11)	0.0244 (11)	0.0021 (9)	0.0127 (9)	−0.0006 (9)
C13	0.0325 (13)	0.0173 (11)	0.0356 (13)	0.0002 (9)	0.0164 (11)	−0.0045 (10)
C14	0.0271 (12)	0.0156 (11)	0.0441 (15)	0.0024 (9)	0.0142 (11)	0.0061 (10)
C15	0.0226 (11)	0.0262 (12)	0.0321 (13)	0.0004 (10)	0.0038 (10)	0.0108 (10)
C16	0.0190 (10)	0.0216 (11)	0.0265 (12)	−0.0010 (9)	0.0032 (9)	0.0029 (9)

### Geometric parameters (Å, º)

**Table:**

Co1—Cl1^i^	2.4070 (5)	C6—C7	1.406 (3)
Co1—Cl1	2.4070 (5)	C7—H7	0.9500
Co1—S1	2.4856 (5)	C7—C8	1.378 (3)
Co1—S1^i^	2.4856 (5)	C9—C4	1.419 (3)
Co1—N1^i^	2.1542 (17)	C9—C8	1.420 (3)
Co1—N1	2.1543 (17)	C10—H10A	0.9900
S1—C8	1.775 (2)	C10—H10B	0.9900
S1—C10	1.832 (2)	C11—C10	1.507 (3)
N1—C1	1.322 (3)	C11—C12	1.394 (3)
N1—C9	1.378 (3)	C11—C16	1.389 (3)
C1—H1	0.9500	C12—H12	0.9500
C2—C1	1.410 (3)	C13—C12	1.393 (3)
C2—H2	0.9500	C13—H13	0.9500
C2—C3	1.362 (3)	C14—C13	1.385 (4)
C3—H3	0.9500	C14—H14	0.9500
C4—C3	1.411 (3)	C14—C15	1.382 (4)
C4—C5	1.417 (3)	C15—H15	0.9500
C5—H5	0.9500	C16—C15	1.394 (3)
C5—C6	1.367 (3)	C16—H16	0.9500
C6—H6	0.9500		
			
Cl1^i^—Co1—Cl1	180.0	C5—C6—H6	119.8
Cl1—Co1—S1^i^	84.016 (17)	C5—C6—C7	120.5 (2)
Cl1—Co1—S1	95.984 (17)	C7—C6—H6	119.8
Cl1^i^—Co1—S1	84.015 (17)	C6—C7—H7	119.5
Cl1^i^—Co1—S1^i^	95.984 (17)	C8—C7—C6	121.0 (2)
S1^i^—Co1—S1	180.0	C8—C7—H7	119.5
N1—Co1—Cl1^i^	88.58 (5)	C7—C8—S1	119.38 (17)
N1^i^—Co1—Cl1^i^	91.42 (5)	C7—C8—C9	119.84 (19)
N1^i^—Co1—Cl1	88.58 (5)	C9—C8—S1	120.78 (16)
N1—Co1—Cl1	91.42 (5)	N1—C9—C4	121.66 (19)
N1—Co1—S1	81.12 (5)	N1—C9—C8	119.66 (18)
N1^i^—Co1—S1^i^	81.12 (5)	C4—C9—C8	118.68 (19)
N1^i^—Co1—S1	98.88 (5)	S1—C10—H10A	110.1
N1—Co1—S1^i^	98.88 (5)	S1—C10—H10B	110.1
N1^i^—Co1—N1	180.00 (9)	H10A—C10—H10B	108.4
C8—S1—Co1	97.58 (7)	C11—C10—S1	108.03 (15)
C8—S1—C10	101.30 (10)	C11—C10—H10A	110.1
C10—S1—Co1	113.32 (8)	C11—C10—H10B	110.1
C1—N1—Co1	121.86 (14)	C12—C11—C10	121.0 (2)
C1—N1—C9	117.47 (18)	C16—C11—C10	119.4 (2)
C9—N1—Co1	120.55 (13)	C16—C11—C12	119.5 (2)
N1—C1—H1	117.8	C11—C12—H12	119.9
N1—C1—C2	124.4 (2)	C13—C12—C11	120.1 (2)
C2—C1—H1	117.8	C13—C12—H12	119.9
C1—C2—H2	120.6	C12—C13—H13	120.0
C3—C2—C1	118.7 (2)	C14—C13—C12	120.0 (2)
C3—C2—H2	120.6	C14—C13—H13	120.0
C2—C3—H3	120.3	C13—C14—H14	119.9
C2—C3—C4	119.4 (2)	C15—C14—C13	120.2 (2)
C4—C3—H3	120.3	C15—C14—H14	119.9
C3—C4—C5	121.6 (2)	C14—C15—H15	120.0
C3—C4—C9	118.32 (19)	C14—C15—C16	120.1 (2)
C5—C4—C9	120.0 (2)	C16—C15—H15	120.0
C4—C5—H5	120.0	C11—C16—C15	120.1 (2)
C6—C5—C4	119.9 (2)	C11—C16—H16	119.9
C6—C5—H5	120.0	C15—C16—H16	119.9
			
Co1—S1—C8—C7	−177.06 (17)	C6—C7—C8—S1	−178.85 (18)
Co1—S1—C8—C9	3.13 (18)	C6—C7—C8—C9	1.0 (3)
Co1—S1—C10—C11	91.07 (15)	C8—S1—C10—C11	−165.48 (15)
Co1—N1—C1—C2	−175.63 (16)	C8—C9—C4—C3	−178.91 (19)
Co1—N1—C9—C4	175.41 (15)	C8—C9—C4—C5	−0.8 (3)
Co1—N1—C9—C8	−5.1 (3)	C9—N1—C1—C2	0.5 (3)
N1—C9—C4—C3	0.6 (3)	C9—C4—C3—C2	−0.2 (3)
N1—C9—C4—C5	178.78 (19)	C9—C4—C5—C6	0.0 (3)
N1—C9—C8—S1	0.5 (3)	C10—S1—C8—C7	67.23 (19)
N1—C9—C8—C7	−179.27 (19)	C10—S1—C8—C9	−112.57 (18)
C1—N1—C9—C4	−0.7 (3)	C10—C11—C12—C13	−178.5 (2)
C1—N1—C9—C8	178.79 (19)	C10—C11—C16—C15	180.0 (2)
C1—C2—C3—C4	−0.1 (3)	C11—C16—C15—C14	−2.1 (4)
C3—C2—C1—N1	−0.1 (3)	C12—C11—C10—S1	64.7 (2)
C3—C4—C5—C6	178.1 (2)	C12—C11—C16—C15	1.2 (3)
C4—C5—C6—C7	1.3 (3)	C13—C14—C15—C16	1.5 (4)
C4—C9—C8—S1	−179.91 (16)	C14—C13—C12—C11	−0.9 (4)
C4—C9—C8—C7	0.3 (3)	C15—C14—C13—C12	0.0 (4)
C5—C4—C3—C2	−178.3 (2)	C16—C11—C10—S1	−114.0 (2)
C5—C6—C7—C8	−1.8 (4)	C16—C11—C12—C13	0.3 (3)

Symmetry code: (i) −*x*+1, −*y*+1, −*z*+1.

### Hydrogen-bond geometry (Å, º)

*Cg*2 is the centroid of the C4–C9 ring.

**Table:**

*D*—H···*A*	*D*—H	H···*A*	*D*···*A*	*D*—H···*A*
C16—H16···*Cg*2^ii^	0.95	2.89	3.575 (2)	131

Symmetry code: (ii) −*x*, −*y*, −*z*.
